# Targeting Delivery System for *Lactobacillus Plantarum* Based on Functionalized Electrospun Nanofibers

**DOI:** 10.3390/polym12071565

**Published:** 2020-07-15

**Authors:** Hongliang Yu, Weihua Liu, Dongmei Li, Chunhong Liu, Zhibiao Feng, Bin Jiang

**Affiliations:** Department of Applied Chemistry, Northeast Agricultural University, Harbin 150000, China; yuhongliang97@163.com (H.Y.); 15561851295@163.com (W.L.); lidongmei@neau.edu.cn (D.L.); liuchunhong@neau.edu.cn (C.L.)

**Keywords:** coaxial electrospinning, polylactic acid, probiotics, nanofibers

## Abstract

With the increased interest in information on gut microbes, people are realizing the benefits of probiotics to health, and new technologies to improve the viability of probiotics are still explored. However, most probiotics have poor resistance to adverse environments. In order to improve the viability of lactic acid bacteria, polylactic acid (PLA) nanofibers were prepared by coaxial electrospinning. The electrospinning voltage was 16 kV, and the distance between spinneret and collector was 15 cm. The feed rates of the shell and core solutions were 1.0 and 0.25 mL/h, respectively. The lactic acid bacteria were encapsulated in the coaxial electrospun nanofibers with PLA and fructooligosaccharides (FOS) as the shell materials. Scanning electron microscopy, transmission electron microscopy, and laser scanning confocal microscopy showed that lactic acid bacteria were encapsulated in the coaxial electrospun nanofibers successfully. The water contact angle test indicated that coaxial electrospun nanofiber films had good hydrophobicity. An in vitro simulated digestion test exhibited that the survival rate of lactic acid bacteria encapsulated in coaxial electrospun nanofiber films was more than 72%. This study proved that the viability of probiotics can be improved through encapsulation within coaxial electrospun PLA nanofibers and provided a novel approach for encapsulating bioactive substances.

## 1. Introduction

Probiotics are defined by the Food and Agriculture Organization of the United Nations and the World Health Organization (FAO/WHO) as living microorganisms that, when administered in adequate amounts, confer a health benefit on the “host” [1]. Probiotics have many functions, including the modulation of microbiota and metabolic activity in the gut, modulation of intestinal motility and absorption, impact on intestinal inflammation, modulation of the immune system and potential modulation of risk factors for cancer development [2]. Probiotics have been widely used in bioengineering, industry and agriculture, food safety, and life health [3,4]. However, most probiotics have poor resistance to adverse environments and are susceptible to temperature, oxygen, solvents, and mechanical oppression during processing. They are also susceptible to gastric acid and bile salts, which considerably reduce the amount of viable bacteria in the human digestive tract. These factors have ultimately made probiotics ineffective [5]. Microencapsulation has been found to be a useful tool for the stabilization of probiotic cells and bioactive substances in functional food applications. It can enhance the viability of probiotic cells during processing, storage, subsequent consumption, and gastrointestinal digestion [6,7]. It has been reported that different methods such as emulsification, phase separation, and spray drying have been used to microencapsulate bioactive substances [7,8,9,10]. However, many of these methods involve high temperatures or a large amount of organic reagents, which may adversely affect the survival rate of probiotics or cause potential hazards to the human body. The electrospinning technique has been widely used to microencapsulate microorganisms and bioactive substances in recent years because it does not require severe temperature and pressure requirements [11,12,13]. Bioactive substances such as folic acid and fish oil have been encapsulated in electrospun fibers [14,15]. Microorganisms such as *Escherichia coli*, *Staphylococcus epidermidis,* and *T4 bacteriophage* have also been encapsulated in electrospun nanofibers [16,17]. Bifidobacterium has been encapsulated in protein (whey protein concentrate)/carbohydrate (pullulan) food hydrocolloids by means of the electrospinning technique [18] and *Lactobacillus rhamnosus* in poly (vinyl alcohol) and sodium alginate-based electrospun nanofibers [19]. Although electrospinning has been proved to be a promising process for encapsulating probiotics, little research has been done in this area. In previous studies, probiotics were encapsulated successfully and the survival rate of probiotics was significantly increased. However, most studies used blended electrospinning with a single base, and functional substances were rarely added to the embedding materials. In addition, many reports have confirmed that the addition of prebiotics can protect the activity of probiotics [20,21,22]. It can be reasonably suggested that adding prebiotics during electrospinning can further improve the survival ability of microencapsulated probiotics [23].

Polylactic acid (PLA) is usually synthesized through lactic acid fermentation. Lactic acid is a type of organic acid that is widely found in animals and plants; therefore, PLA has good biocompatibility [24]. PLA can be degraded in the human body, and the degradation product is directly metabolized by the human body to give the final products CO_2_ and H_2_O, which can be excreted out of the body through the kidneys. The US Food and Drug Administration (FDA) has long approved PLA use for absorbable surgical sutures, injection capsules, orthopedic fixation materials, microspheres for drug release, and tissue-engineered stents [25]. At present, PLA is considered as one of the most important materials with biodegradability and is widely used in the field of biomedicine [26,27].

In this study, the probiotics represented by lactic acid bacteria were encapsulated in PLA nanofibers by coaxial electrospinning, and fructooligosaccharides (FOS) were added as a prebiotic to improve the viability of probiotics in the digestive system. The physicochemical properties of the electrospun nanofibers were analyzed by means of scanning electron microscopy (SEM) micrographs, Fourier transform infrared spectroscopy (FT-IR), water contact angle, and differential scanning calorimetry (DSC). The survival rate of probiotics was studied via in vitro simulated digestion experiments.

## 2. Materials and Methods

### 2.1. Materials

PLA (4032D) was purchased from NatureWorks LLC. (Minnetonka, MN, USA). Dichloromethane (DCM) and *N,N*-Dimethylformamide (DMF) were obtained from TianJin Fine Chemicals Co., Ltd. (TianJin, China). Disodium hydrogen phosphate (Na_2_HPO_4_) and potassium dihydrogen phosphate (KH_2_PO_4_) were purchased from Aladdin Reagent Co., Ltd. (Shanghai, China). Fructooligosaccharides (FOS), Rhodamine 123, and de Man−Rogosa−Sharpe (MRS) medium were obtained from Beijing Solarbio Science & Technology Co., Ltd. (Beijing, China). Lactic acid bacteria (*Lactobacillus plantarum* 1.0665) were donated by the College of Food Science, Northeast Agricultural University (Harbin, China).

### 2.2. Electrospinning Solution Preparation

The shell solution was prepared by dissolving PLA in a mixture of DCM and DMF with a volume ratio of 7:3, and then FOS was added. The shell solution was magnetically stirred at room temperature (20–25 °C) for 6 h until it was dissolved completely. The concentration of PLA was 8% (*w*/*v*), and the concentration of FOS was 2.5% (*w*/*v*). The core solution was prepared by inoculating 1% (*v*/*v*) *L. plantarum* 1.0665 into the MRS medium [23], which was cultured at 37 °C for 24 h. The concentration of the lactic acid bacteria was determined by the standard plate count and diluted to 10^8^ CFU/mL.

### 2.3. Electrospinning

Electrospinning was performed using a high-voltage direct power supply, and the shell and core solutions were consecutively filled in a 10-mL plastic syringe. A stainless steel needle with an inner diameter of 0.84 mm was used in shell electrospinning. A stainless steel needle with inner and outer diameters of 0.34 and 1.01 mm, respectively, was used in coaxial electrospinning. The feed rates of the shell and core solutions were 1.0 and 0.25 mL/h, which were controlled by syringe pumps. The electrospinning voltage was 16 kV, and the distance between spinneret and collector was 15 cm. The obtained electrospun nanofibers were collected using a grounded plate covered with a tin foil, and the resulting nanofiber films were stored in sterile boxes. The electrospinning process was carried out at 20–25 °C, and the relative humidity was around 40–60%.

### 2.4. Viscosity of Shell Solution

The viscosity of shell solution was measured by a Brookfield digital viscometer (DV-S, AMETEK. Inc., San Diego, CA, USA) with an s61 rotor at 20 °C. The depth of the immersion solution was based on the groove above the probe [28]. The data were recorded when the number was stable.

### 2.5. Scanning Electron Microscopy (SEM)

The electrospinning nanofiber films were cut out and adhered to the sample stage with a conductive tape. After sputtering Au and carbon coating with an ion sputtering apparatus, the microscopic morphology of the nanofibers were observed using a scanning electron microscope (SU 8010, Hitachi, Ltd., Tokyo, Japan); the acceleration voltage was 5 KV. The diameter of the nanofibers was measured using Image-Pro Plus software. The average diameter of nanofibers was calculated from the obtained data and plotted as a diameter distribution histogram.

### 2.6. Fourier Transform Infrared Spectroscopy

The electrospun nanofiber films and the materials were analyzed using a FT-IR spectrometer (iS50, Thermo Fisher Scientific Inc., Waltham, MA, USA) with an Attenuated Total Reflection (ATR) device. The electrospinning nanofiber films were cut into 3 cm × 3 cm sheets and placed on the ATR device, and the materials were measured using a KBr disk with spectral resolution of 4 cm^−1^, wavenumber range of 4000–400 cm^−1^, and 64 scans [29].

### 2.7. Differential Scanning Calorimetry

The thermal behavior of the electrospun nanofiber films and materials was analyzed using a differential scanning calorimeter (Q2000, TA Instruments, New Castle, DE, USA). Accurately weighed samples of 6–10 mg were placed in sealed aluminum crucibles. An empty crucible under the same conditions was used as a reference. The samples were heated from 20 to 230 °C with a heating rate of 10 °C/min under a nitrogen atmosphere.

### 2.8. Water Contact Angle

The water contact angles of the electrospun nanofiber films were measured using a water contact angle meter (XG-CAM, Shanghai Sunzern Instrument Co., Ltd., Shanghai, China). The electrospun nanofiber films were cut into 10 cm × 10 cm sheets and fixed to glass slides. A droplet of water was added to the surface of the electrospinning nanofiber films. The averages of five measurements recorded at different areas of each sample were taken as the water contact angles. The water contact angles were measured from 0° to 180° with an accuracy of ± 0.1°. The Young–Laplace equation was used to calculate the water contact angles.

### 2.9. Lactic Acid Bacteria Embedding Analysis

#### 2.9.1. Transmission Electron Microscopy (TEM)

The coaxial electrospun nanofibers were collected directly onto the copper grids. The morphology and core/shell structure of the coaxial electrospinning nanofibers were observed by using a transmission electron microscope (H-7650, Hitachi Ltd., Tokyo, Japan). The images were captured using an Olympus imaging system [30].

#### 2.9.2. Laser Scanning Confocal Microscopy (LSCM)

The lactic acid bacteria were stained by Gram stain and observed using an inverted light microscope. The presence and distribution of lactic acid bacteria stained with Rhodamine 123 were observed using an inverted laser scanning confocal microscope (TCS SP8, Leica Microsystems GmbH, Wetzlar, Germany) with an excitation wavelength of 488 nm to check whether the lactic acid bacteria were properly encapsulated within the coaxial electrospinning nanofibers [31,32].

### 2.10. In Vitro Digestion

The in vitro digestion studies were carried out and improved following the methodology proposed by Marysol Aceituno-Medina, et al. [33]. Coaxial electrospun nanofiber films (20 mg) were added in 20-mL simulated gastric fluid (pepsin 3.2 g/L, NaCl 0.2 g/L, pH = 1.2) and shaken in a 37 °C water bath for 2 h. Subsequently, the coaxial electrospinning nanofiber films after in vitro simulated gastric digestion were added in 20 mL simulated intestinal fluid (trypsin 10.0 g/L, KH_2_PO_4_ 6.8 g/L, pH = 7.0) and incubated at 37 °C for 2 h. The digestive fluid was taken out, and the concentration of the lactic acid bacteria was measured by the standard plate count. Undigested coaxial electrospun nanofiber films under the same conditions were used as a control, the concentration of which was considered as 100%.

### 2.11. Statistical Analysis

Experiments were performed in triplicate. Statistical analysis was performed using SPSS Statistics Software (20.0, Chicago, IL, USA). The obtained data are presented as the mean ± standard deviation.

## 3. Results and Discussion

### 3.1. Solution Properties

Three conditions should be satisfied for a solution to produce a stable jet in an electrostatic field. Firstly, there should be the ability to generate inductive charge, which causes the charged jet to form a wave back and to form the spinning body in the electrostatic field. Secondly, the solvent should have a good solubility for the solute to ensure that solute molecules in the solvent are evenly dispersed and at the receiving end to form a spinneret. Finally, the solvent system can be composed of multiple solvents that are volatile [34,35]. After the solvent is charged, it completely volatilizes between the transmitting end and the receiving end. When the solvent volatilization degree is low, the nanofibers collected at the receiving end are affected by solvent destruction, resulting in adhesion and deformation of the spinning body. The solubility parameter of PLA (δ1) is about 20 J^1/2^/cm^3/2^, which is equivalent to that of non-polar materials. Chloroform, dichloromethane, tetrahydrofuran and others could be used as good solvents for PLA. However, DCM has a relatively lower toxicity than the aforementioned solvents. The conductivity of DCM can be increased by mixing DMF with high charge induction capability to stabilize the jet flow. The solubility parameter of the mixed solvent system (δ2) is 17.9~19.85 J^1/2^/cm^3/2^, 0.35 ≤| δ1 − δ2 |≤ 3.9. Therefore, the mixed solvent could be used as a good solvent for PLA electrospinning.

**Figure 1:**
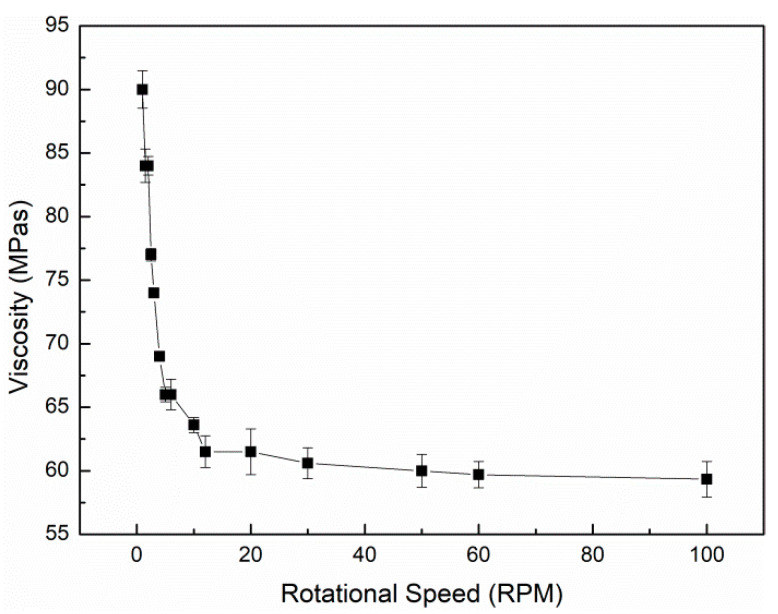
Relationship between the viscosity of the shell solution and the rotational speed at 20 °C. Shell solution: polylactic acid (PLA, 8%, *w*/*v*) and fructooligosaccharides (FOS, 2.5%, *w*/*v*) dissolved in the mixed solution of dichloromethane (DCM) and *N*,*N*- Dimethylformamide (DMF) (7:3, *v*/*v*).

The viscosity of the electrospun solution directly affects the morphology and properties of the nanofibers obtained by electrospinning. The higher the viscosity of the solution, the more easily the polymer molecular chain will become entangled and the more unstable the jet flow will be, which makes the electrospinning process more difficult and it is not easy to produce nanofibers with a uniform diameter distribution. However, when the viscosity of solution is low, the solution cannot form a jet in the electrostatic field, but only form microdroplets. Therefore, the key of electrospinning is to prepare a solution with appropriate viscosity [36]. The viscosity of the shell solution is shown in Figure 1. The rotor of a Brinell viscometer was driven by the motor to rotate at a constant speed by changing speed. When the rotor rotated in the solution, the solution generated a viscous torque on the rotor. The greater the viscosity of the solution, the greater the viscous torque and vice versa. Figure 1 shows that the viscosity of shell solution decreases with an increase in the rotational speed, which was in agreement with the shear thinning characteristic of non-Newtonian fluid.

### 3.2. Scanning Electron Microscopy Analysis

**Figure 2:**
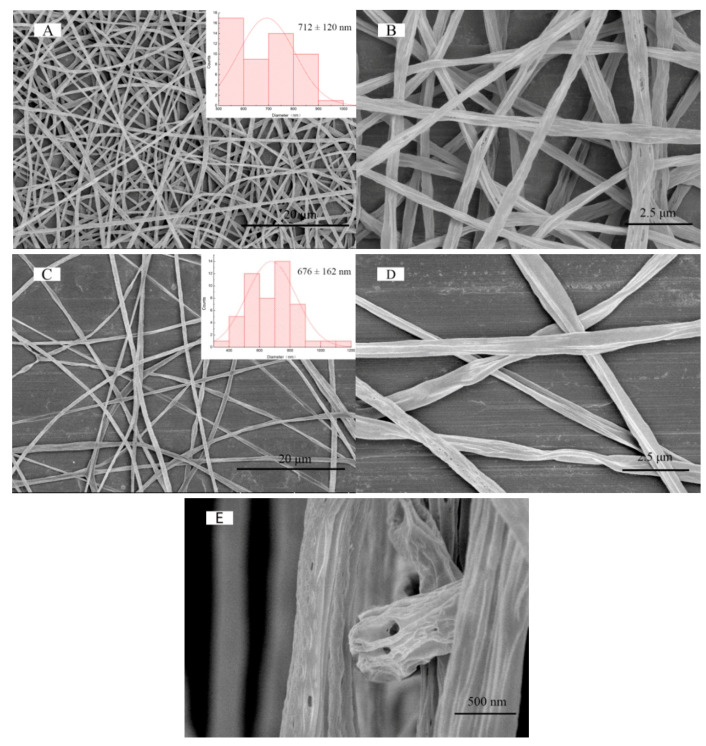
Scanning electron microscopy (SEM) micrographs at different magnifications of (**A**,**B**) shell and (**C**,**D**) coaxial electrospun nanofibers; and (**E**) the cross-section of coaxial electrospun nanofibers.

The microstructure of the shell and coaxial electrospinning nanofibers were analyzed by scanning electron microscopy. As shown in Figure 2A,C, the diameters of the shell and coaxial electrospun nanofibers are 712 ± 120 and 676 ± 162 nm, respectively. As demonstrated in Figure 2B,D, “holes” appeared on the surface of electrospun nanofibers due to different rates of solvent evaporation (DMF and DCM) [37,38]. In the coaxial electrospinning system, since the evaporation of the core solvent was lower than that of the shell solvent, the jet generated cracking “branches” in the electric field and the coarser primary nanofibers were split to form fine nanofibers with a smaller diameter. When the solidified polymer was in an electrostatic field, its amorphous parts formed a semi-solid state of “wet fiber” approximately under the action of electric field and ambient temperature, which might form a Taylor cone on the surface and then stretch again in a short period of time [39]. This might be one of the reasons why the diameters of coaxial electrospun nanofibers were smaller than those of shell electrospun nanofibers. The typical hollow structure of coaxial electrospun nanofibers is shown in Figure 2E.

### 3.3. FT-IR Analysis

**Figure 3:**
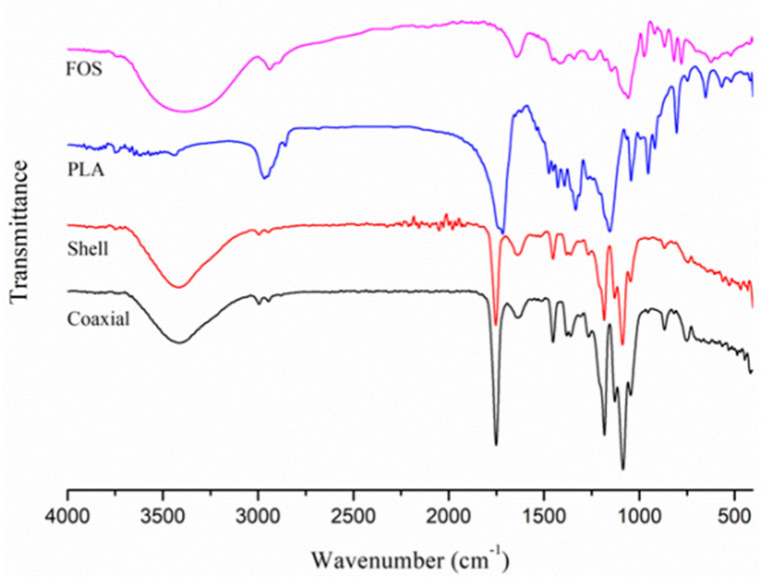
Fourier transform infrared (FT-IR) spectra of PLA, FOS, shell, and coaxial electrospun nanofiber films.

The FT-IR spectra of PLA, FOS, shell and coaxial electrospun nanofiber films are shown in Figure 3. The spectra of original PLA had no characteristic absorption peak at 3000–4000 cm^−1^, while the spectra of FOS had characteristic absorption peak (O–H stretching) at 3385 cm^−1^. Similarly, the absorption peaks (O–H stretching) of shell and coaxial electrospinning nanofiber films occur at 3410/3420 cm^−1^, indicating that FOS was successfully electrospun into the shell nanofibers. The carboxyl group in PLA caused the O–H stretching peak of the shell and coaxial electrospinning nanofiber films to exhibit a blue-shift. The spectra of PLA possessed peaks at 2967/2857 cm^−1^ (CH_3_ inverse/symmetric), 1733 cm^−1^ (C=O), 1154 cm^−1^ (C–O–C), and 1045 cm^−1^ (C–C). The spectra of FOS showed peaks at 2939/2902 cm^−1^ (CH_3_ *inverse*/*symmetric*), 1146 cm^−1^ (C–C), and 1058 cm^−1^ (C–O–C). Most of the peaks of PLA and FOS with different strengths were found in the spectra of shell and coaxial electrospun nanofiber films, indicating that both the substances exist in the shell electrospun nanofibers.

### 3.4. Differential Scanning Calorimetry

**Figure 4:**
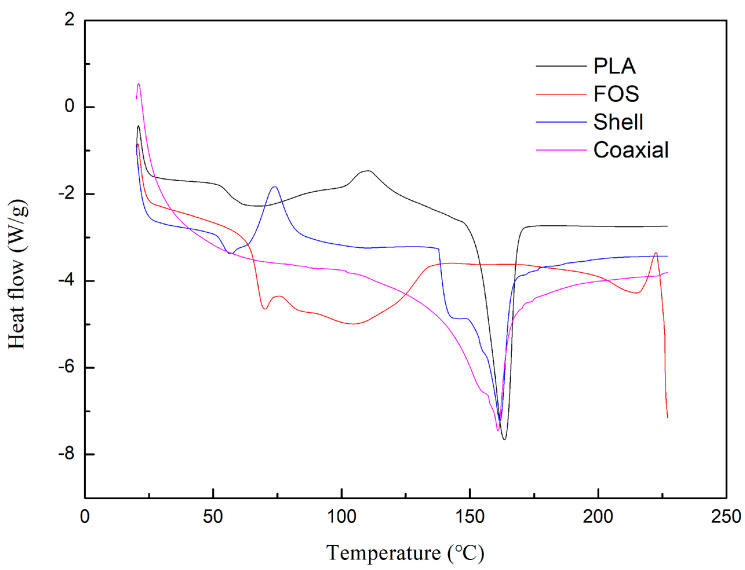
Differential scanning calorimetry (DSC) measurements of PLA, FOS, shell, and coaxial electrospinning nanofiber films.

The viscoelastic and viscoplastic properties of polymers are related to their melting and glass transition temperatures, respectively. PLA is a semi-crystalline polymer containing both amorphous and crystalline domains; therefore, it shows both glass transition and melting temperatures [40]. The results of the DSC measurements are shown in Figure 4. The curve of PLA indicated that the glass transition temperature and melting point are 65 and 163 °C, respectively. Melting is an endothermic process, and crystallization is an exothermic process. The cold crystallization temperature (Tcc) of PLA was about 110 °C. The DSC curve of shell electrospinning nanofiber films showed that the glass transition temperature was slightly different from that of PLA, which was 63 °C. In fact, the glass transition temperature of FOS was 69 °C. However, it was possible that due to the interaction between PLA and FOS, the crystal structure was destroyed and the glass transition temperature of shell electrospinning nanofiber films was depressed [41]. The DSC curve of coaxial electrospinning nanofiber films only showed an obvious melting temperature of 161 °C. This might be due to the residual water molecules in the core of coaxial electrospinning nanofibers. The peak value of melting point on the curve indicated that the effect of electrospinning on melting point is not significant.

### 3.5. Water Contact Angle

**Figure 5:**
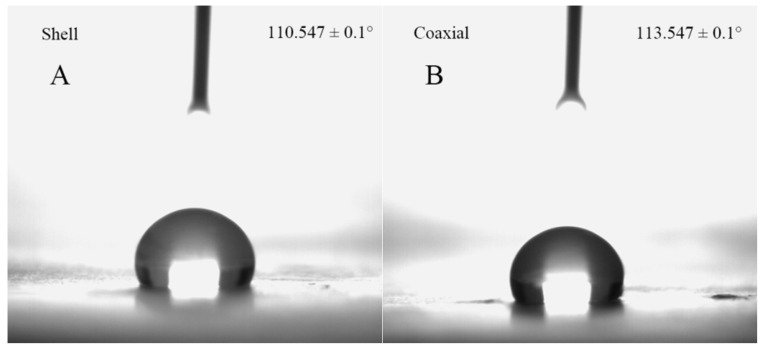
Water contact angles of (**A**) shell and (**B**) coaxial electrospun nanofiber films.

Hydrophobicity is an important parameter of microcapsule encapsulation materials, which is usually characterized by the water contact angle. In general, a material is called hydrophilic when the intrinsic water contact angle is smaller than 90°, and a material is called hydrophobic when the intrinsic water contact angle is greater than 90° [42]. As shown in Figure 5, the water contact angles of the shell and coaxial electrospun nanofiber films were 110.5 ± 0.1° and 113.5 ± 0.1°, respectively. Similar results for PLA have been reported in other studies [43,44]. The hydrophobicity of PLA is related to its methyl group. Coaxial electrospinning did not significantly affect the hydrophobicity of PLA. The strong hydrophobicity of the PLA coaxial electrospun nanofibers films is an attractive feature for microcapsule encapsulation technologies.

### 3.6. Lactic Acid Bacteria Embedding Analysis

**Figure 6:**
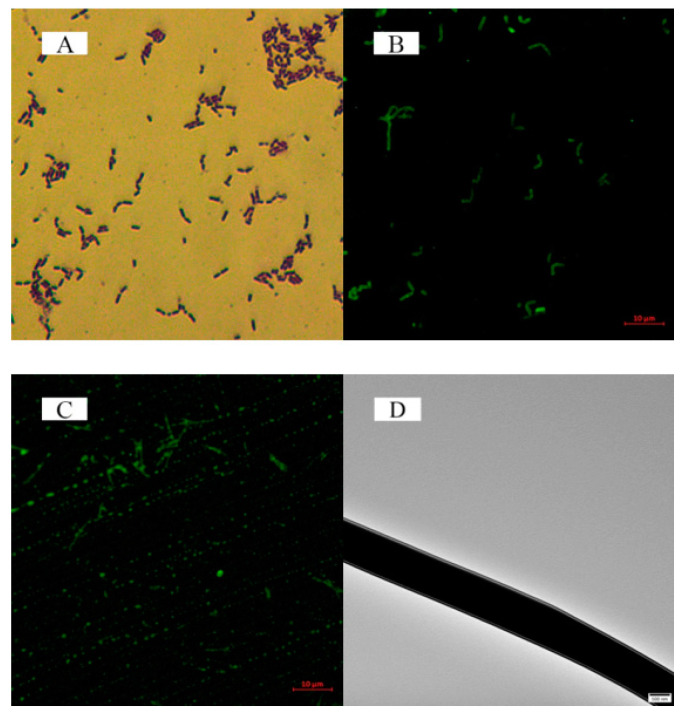
(**A**) Inverted optical microscope image of lactic acid bacteria, (**B**) Laser scanning confocal microscopy (LSCM) image of lactic acid bacteria, (**C**) LSCM image of coaxial electrospun nanofibers, and (**D**) Transmission electron microscopy (TEM) image of coaxial electrospun nanofibers.

Inverted optical microscope images of lactic acid bacteria and laser scanning confocal microscope images of lactic acid bacteria and coaxial electrospun nanofibers were captured. Figure 6A,B indicates that lactic acid bacteria are distributed randomly in the MRS medium. Figure 6C shows that the lactic acid bacteria are in a linear arrangement, indicating that the lactic acid bacteria were successfully encapsulated in the coaxial electrospun nanofibers. The core/shell structure of the coaxial electrospun nanofibers was analyzed by trasmission electron microscopy (TEM). The analysis proved that the MRS medium containing lactic acid bacteria was confined in the coaxial electrospun nanofibers, as shown in Figure 6D.

### 3.7. Lactic Acid Bacteria In Vitro Simulated Digestion

The food needs to be digested in the stomach across four stages for about 2 h. The free lactic acid bacteria without encapsulation decreased rapidly after the simulated digestion for 0–2 h, and the number of viable bacteria decreased to zero [45]. The electrospinning nanofibers should not be dissolved or broken during this period to ensure that the lactic acid bacteria are not digested under the conditions of high acidity and gastric digestive enzymes so that the intestine can be smoothly reached. PLA is well-known to have good acid resistance [46], and experimental results have also shown that the coaxial electrospun nanofiber films remain stable in the gastric acid environment. However, in the simulated intestinal fluid, with the slow dissolution of the shell electrospun nanofiber films, lactic acid bacteria in the core were found to release gradually. The viable count of lactic acid bacteria contained in the coaxial electrospun nanofiber films was about 1.1 × 10^4^ CFU/mg. Under the condition of simulating digestion of intestinal solution for 2 h, the viable count of lactic acid bacteria was 8 × 10^3^ CFU/mg, which remained above 72%. According to the FT-IR results, FOS was contained in shell electrospun nanofibers, which could selectively promote the further growth of lactic acid bacteria and improve their activity. Yoha et al. [47] used spray drying (SD) and spray freeze drying (SFD) techniques to encapsulate L. plantarum (NCIM 2083). Under in vitro simulated digestion, free probiotic cells were found to lose their viability. Under small intestinal conditions, 4 log reductions in the cell viability of SD synbiotics were observed. SFD synbiotics exhibited a final viability loss of 2 log reductions. Ma et al. [48] prepared probiotic microcapsules using an optimized emulsification method. The survival rate of probiotic microcapsules after 90 min of exposure to simulated gastric fluid was 53.3%, which was significantly higher than that of free cells (16.81%; *p* < 0.5). The above discussion confirms that electrospinning is a successful microencapsulation technique. However, there are a few limitations that will hinder the progress of its applications. For instance, in the food industry, the biocompatibility of materials is necessary. In order to utlize electrospun products prepared in laboratory for practical applications, further research is required to have an in-depth understanding of the materials and address the limitations [49].

## 4. Conclusions

In this study, coaxial PLA electrospun nanofibers containing *L. plantarum* were prepared. The average diameter of the coaxial electrospun nanofibers was 676 ± 162 nm. As observed in the LSCM image, the lactic acid bacteria exhibited a linear arrangement, indicating that the bacteria were successfully encapsulated in the coaxial electrospun nanofibers. In addition, the DSC and FT-IR analysis of the coaxial electrospun nanofiber films showed the characteristics of PLA, FOS, and nanofiber films, which provided further evidence for the successful preparation of coaxial electrospun nanofibers. The water contact angle of coaxial electrospun nanofiber films was 113.5 ± 0.1°, thus indicating their hydrophobic nature. Moreover, the in vitro simulated digestion of lactic acid bacteria showed that during digestion, the number of viable bacteria remained above 72%. Therefore, with PLA and fructooligosaccharides (FOS) as shell materials and by encapsulating lactic acid bacteria using the coaxial electrospinning technique, the nanofiber films were found to exhibit good hydrophobicity and biocompatibility, and the encapsulated lactic acid bacteria showed good activity. This study provides a novel approach to the encapsulation of bioactive substances.
